# Extensive perineal hidradenitis suppurativa and concurrent lymphedema treated with radical surgical excision and perineal reconstruction

**DOI:** 10.1016/j.jdcr.2025.06.008

**Published:** 2025-06-19

**Authors:** Aaron Cheng, Armand Berry, Payden Harrah, Thomas Lee, Victoria G. Farley

**Affiliations:** a Long School of Medicine, University of Texas Health San Antonio, San Antonio, Texas; b Valley Health Physician Alliance - Plastic Surgery, Las Vegas, Nevada; c Vivida Dermatology, Las Vegas, Nevada; d University of Nevada Las Vegas School of Medicine, Las Vegas, Nevada

**Keywords:** hidradenitis suppurativa, lymphedema, perineal reconstruction

## Introduction

Hidradenitis suppurativa (HS) is a rare chronic and debilitating inflammatory skin condition that typically presents as painful deep-seated nodules, abscesses, skin tunnels, and fibrotic scars in intertriginous areas.^1^^,^^2^ The most commonly affected areas are the axillary, groin, perianal, and inframammary regions leading to severe physical pain that frequently restricts physical activity.^3^^,^^4^ Diagnosis can be challenging due to its variable presentation, delayed recognition, and intermittent symptoms often leading to exacerbating the patient experience.^5^ Treatment varies based on disease severity, ranging from topical antibiotics to surgical interventions.^6^ However, the primary goals are to relieve lesion-related symptoms while preventing disease progression and recurrence.^6^ Herein, we report a unique case of extensive HS of the perineum and vulva with concurrent severe lymphedema managed surgically with radical excision and perineal reconstruction.

## Case report

**Fig 1:**
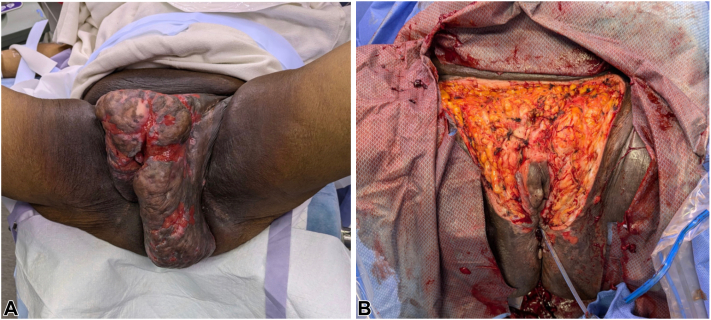
**A,** Clinical preoperative photograph exhibiting large, pedunculated lesions with exposed, ulcerated, and erythematous skin around the mons pubis, and bilateral labia majora of the vulva. **B,** Clinical intraoperative photograph of defect post removal of specimen with a preserved margin of skin around the clitoris and labia.

A 64-year-old Black female with a history of HS, managed with 80 mg subcutaneous adalimumab every 2 weeks, presented with a worsening flare involving the mons pubis and vulva, along with a growing mass (Fig 1, *A*). The patient reported severe pain, drainage, and bleeding in the entire region and stated that it had worsened and grown in the past 6 months. Despite treatment with oral doxycycline, cefdinir, spironolactone, metformin, and infliximab, her symptoms persisted. Interleukin-17 inhibitors were contraindicated due to her history of inflammatory bowel disease. She previously underwent surgical excision for the same issue 3 years ago, which provided temporary relief.

On physical examination, a large ulcerated edematous mass with open full thickness skin wounds with active discharge and areas of new epithelialization involving the perineum, vulva, and mons pubis consistent with Hurley Stage III HS was observed (Fig 1, *A*). The labia majora were also enlarged bilaterally with edema and erythematous, ulcerated skin (Fig 1, *A*). Biopsy of the lesion was consistent with HS, ruling out cutaneous neoplasms. The patient reported severe pain, drainage, and bleeding in the entire region and stated that it has worsened and grown in the past 6 months.

Due to the extensive involvement and severity of the disease, she was planned for full thickness skin excision under general anesthesia with staged reconstruction following subsequent hospital admission for local wound care. Local anesthesia consisting of 1% lidocaine with epinephrine was infiltrated subcutaneously at the planned incision for analgesia and hemostasis. The skin was incised full thickness sharply. The Enseal, an advanced bipolar dissecting tool, was used to dissect through the subcutaneous fat. The specimen was completely excised, preserving a margin of skin around the clitoris and labia majora (Fig 1, *B*). The remaining defect measured 30 × 20 cm in greatest dimensions. Hemostasis was obtained with electrocautery, and the wound was dressed with xeroform and a dry bulky dressing.

**Fig 2:**
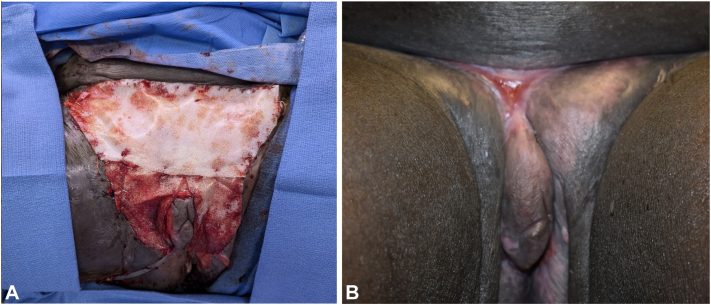
**A,** Clinical intraoperative photograph of defect with Polynovo BTM covering the vagina and mons pubis. **B,** Two-week follow up clinical image showing the defect mostly healed with some granulation and a small 2 cm lateral open wound at the left labial fold and a 1 cm open wound at the right upper pubic ridge along the fold between the panniculus and mons pubis.

Two weeks later, the patient returned for perineal reconstruction. The wound was inspected and showed areas with an exudative layer. These areas were debrided to healthy bleeding granulation tissue and new wound edges were created around the vagina. The wound measured 20 × 16 cm in greatest dimensions. A 10 × 10 cm piece of Polynovo Biodegradable Temporizing Matrix was cut to fit the defect around the vagina. A 20 × 10 cm piece of Polynovo Biodegradable Temporizing Matrix was cut to fit the upper defect including the mons. Both were secured to the wound edges with 3-0 Vicryl suture (Fig 2, *A*). The wound was then dressed with xeroform and a bulky dry dressing.

Four weeks after the initial excision, removal of the outer layer of Biodegradable Temporizing Matrix showed a healthy layer of uniform granulation and new epithelialization along the wound edges. The wound was mostly healed with some granulation and a small 2 cm lateral open wound at the left labial fold and a 1 cm open wound at the right upper pubic ridge along the fold between the panniculus and mons pubis (Fig 2, *B*). No signs of irritation, erythema, or infection were noted. At her 2-week follow up appointments, the patient continued to do well with no complaints. She was managed by dermatology and plastic surgery and remained on adalimumab for HS.

## Discussion

HS is a chronic inflammatory skin condition caused by pilosebaceous unit occlusion and immune dysregulation.^1^^,^^2^ HS can be debilitating for patients as it commonly results in recurrent painful abscesses, odor, scarring, and involvement of sensitive areas severely impacting patients’ quality-of-life.^7^ Advanced HS can result in scarring which may restrict limb mobility and interfere with lymph drainage further exacerbating preexisting edema as seen in our case.^8^

Treatment varies depending on disease severity according to Hurley staging, affected areas, patient comorbidities, and response to previous therapies.^1^ However, in complex cases of HS, it is important to consider an individualized, multidisciplinary approach as this disease has an extremely heterogeneous clinical presentation.^9^ Although there is no current consensus on the utility and timing of surgical management for HS, it is recommended in patients who fail medical therapies and those with debilitating disease involvement.^8^

For this case, wide surgical excision and reconstruction were useful due to the severity of the patient's disease. Despite appropriate medical treatment, our patient was experiencing progressive disease recurrence which significantly impacted her quality-of-life. Additionally, previous surgical excisions of the lesion may have resulted in disruption of the lymphatics to the lower mons and bilateral labia, causing swelling on top of active HS. This swelling further exacerbated her symptoms worsening her overall disease burden. The chronic pain, skin maceration, and difficulty with hygiene made resection necessary to restore function and improve overall outcomes. Notably, she remained on adalimumab throughout her surgical course, which is associated with improved outcomes when combined with wide excision surgery.^10^ Close follow-up with dermatology and plastic surgery are necessary to ensure a favorable prognosis.

In conclusion, a multidisciplinary evaluation of advanced stage HS is crucial to select the most appropriate treatment modality. Plastic surgery played an essential role in this case, with timely treatment to remove diseased tissue, shared decision-making, and to help prevent recurrence. Reconstruction techniques, adequate wound therapy, and proper follow-up from multiple care teams is vital for ensuring a comprehensive approach tailored to the patient’s needs, significantly enhancing patient care.

## Conflicts of interest

Dr Farley: Abbvie-principal investigator, speaker, and advisory boards; Sun-principal investigator; Acrotech-principal investigator; Allakos-principal investigator; Amgen-principal investigator; Anaptys Bio-principal investigator; Janssen-principal investigator; Moonlake-principal investigator; Novartis-principal investigator; Pfizer-advisory board; Sanofi-principal investigator; Takeda-principal investigator; UCB- speaker. Authors Cheng, Berry, Harrah, and Lee have no conflicts of interest to declare.
